# Evaluating How Different Drying Techniques Change the Structure and Physicochemical and Flavor Properties of *Gastrodia elata*

**DOI:** 10.3390/foods13081210

**Published:** 2024-04-16

**Authors:** Rong Ma, Hao Cheng, Xinyao Li, Guoquan Zhang, Jianmei Zheng

**Affiliations:** Shaanxi Union Research Center of University and Enterprise for Grain Processing Technologies, College of Food Science and Engineering, Northwest A&F University, Yangling, Xianyang 712100, China; 15121878997@163.com (R.M.); 090611mhm.1314@gmail.com (H.C.); lixinyao202302@163.com (X.L.); zhanggq98@126.com (G.Z.)

**Keywords:** *G. elata*, drying techniques, physicochemical properties, flavor component

## Abstract

We evaluated the drying characteristics and structure, as well as the physicochemical and flavor properties, of *G. elata* treated by hot-air drying (HAD), vacuum drying (VD), freeze drying (FD), microwave drying (MD), and microwave vacuum drying (MVD). We found that MD and MVD showed the shortest drying times, while FD and MVD were able to better retain the active ingredients and color of the samples. However, the different drying methods did not change the internal structure of *G. elata*, and its main components did not fundamentally change. In addition, E-nose and HS-SPME-GC-MS effectively differentiated the volatile components, and 36 compounds were detected by HS-SPME-GC-MS. Of these samples, alcohols and aldehydes were the main substances identified. In particular, MVD samples possessed the most species of organic volatiles, but the FD method effectively eliminated pungent odors from the *G. elata*. Overall, MVD shows the most obvious advantages, improving drying rate while maintaining the original shape, color, and active components in *G. elata*. Ultimately, MVD is the preferred method to obtain high-quality dried *G. elata*, and our drying-method characterizations can be used to investigate similar structural and chemical changes to similar herbs in the future.

## 1. Introduction

Tianma is a dried tuber from the orchid family that is formally classified as *Gastrodia elata* Blume (*G. elata*) and is mainly distributed in East Asia in areas such as China, Korea, Japan, and India [1]. Typically, it acts as a traditional Chinese herb that has been found to have value as a medicine and health food. *G. elata* mostly contains phenols, polysaccharides, organic acids, sterols, and other components [2], among which Gastrodin, Gastrodia polysaccharide, p-hydroxybenzyl alcohol, p-hydroxybenzaldehyde, and balisin are the main active substances. Together, the compounds have the potential to produce sedative and neuroprotective effects [3], as well as antioxidative [4] and anti-inflammatory changes [5] that can protect against Alzheimer’s disease [6]. Since 2019, *G. elata* has become a legal “homologous drug food” species, which has increased the development of *G. elata* health food and enhanced its production and cultivation in China.

Traditionally, Chinese medicines are pretreated by de-enzyming. During the process, the β-glucosidase in fresh *G. elata* is inhibited by decomposing gastrodin [7]. The de-enzyming treatment typically entails boiling and steaming herbs in several ways [8]. Previously, Ma et al. [7] found that the bioactivity achieved by steaming *G. elata* was significantly superior to that of unsteamed *G. elata* due to the reversal of amino acid-related metabolites. Likewise, Wu et al. concluded that steaming combined with HAD (hot air drying) or FD (freeze drying) is a very promising approach to achieve higher bioactivity in *G. elata* processing. In addition, Xie et al. [9] investigated the effect of high-humidity hot air impingement steaming on the physicochemical properties of *G. elata* and found that *G. elata* could be steamed at starch-gelatinization temperatures. In summary, steaming will change the tissue structure of *G. elata*, and the drying and water-loss processes in *G. elata* after steaming are different from that of fresh *G. elata*. However, it is still not clear how the drying characteristics change the physical and chemical structure of *G. elata,* and there is not much research concerning the drying and steaming effects on the quality and flavor of *G. elata*.

Typically, *G. elata* is dried in a baking room with drying conditions and parameters that are not standardized. This ultimately causes the intrinsic quality of *G. elata* to not be unified to a standard of judgment. Likewise, processing operators only dry by experience, so no quantitative data have been collected. Thus, drying efficiency is low, and the product quality is inconsistent and poor due to the serious loss of active ingredients. In industry, the drying process for *G. elata* mainly consists of hot-air drying (HAD), vacuum drying (VD), freeze drying (FD), and other conventional drying methods, which significantly affects the quality of *G. elata* [10]. In addition to these techniques, emerging high-quality drying technologies, such as microwave vacuum drying (MVD), have also been applied in the context of food and agricultural products, especially shiitake mushrooms [11], Tremella fuciformis [12], mint leaves [13], Scutellariae Radix [14], and licorice [15]. MVD combines the advantages of both microwave drying and vacuum drying, and allows the sample to absorb microwaves to generate heat that is transmitted from the inside to the outside to create a pressure gradient that rapidly dehydrates samples. During the process, a large number of bubbles are generated inside the sample, which are conducive to the evaporation of water and the formation of a crunchy texture [16]. At the same time, the loss of the active ingredients in the sample is negligible, which produces a short and efficient drying time. Still, there are almost no studies that have investigated whether microwave vacuum drying results in physical and chemical changes to *G. elata*.

In this study, our goal was to compare the effects of different drying techniques, including hot-air drying (HAD), vacuum drying (VD), freeze drying (FD), microwave drying (MD), and microwave vacuum drying (MVD), on the chemical and physical characteristics of *G. elata*, as well as on its flavor profile and active ingredients, to determine the optimal method for drying *G. elata*. At the same time, the specific effects of steaming on the drying of *G. elata* were explored to provide a theoretical basis for the pretreatment of *G. elata*.

## 2. Materials and Methods

### 2.1. Material

Black and red hybrid *G. elata* was harvested from Ningqiang County, Shanxi Province, China. All *G. elata* samples were stored at 4 ± 1 °C with 85 ± 5% relative humidity in a refrigerator after harvest. In order to kill the active enzyme, the herb is usually pretreated by steaming. Since the size of the *G. elata* affects the steaming time, fresh *G. elata* is cleaned and dried and graded, with samples more than 150 g as the first grade, those between 70–150 g as the second grade, and samples less than 70 g as the third grade. Keeping the water in the steamer at a constant boil, the *G. elata* was placed in a compartment to be steamed using water vapor. The first grade was steamed for 35 min, the second grade was steamed for 30 min, and the third grade was steamed for 25 min. In this process, the *G. elata* changes from raw to cooked. The *G. elata* samples became transparent after the completion of steaming, and there was no white heart in the cross-section. After they cooled to room temperature, the samples were cut into cylindrical slices with a thickness of 2 mm. It is important to note that *G. elata* samples were harvested from the same batch.

### 2.2. Dried G. elata

The following five different drying methods were used to dry 30 g *G. elata* from each sample. The initial moisture content of fresh *G. elata* was 78.4 ± 1.4%. The moisture content during the drying process continuously recorded using an analytical balance (Sartorius, TE214S, Goettingen, Germany). The drying time was determined when the moisture content of the samples was less than 15%. The dried *G. elata* slices were then pulverized with a pulverizer (2000Y, Western Kitchen Power Co., Ltd., Ningbo, China). The prepared *G. elata* powder was passed through a 60 mesh sieve.

HAD: *G. elata* slices were evenly spread on a tray, and then placed in the hot-air-drying oven (FED720, Binder, Neckarsulm, Germany). The drying temperature was set to 60 °C and the wind speed was 0.2 m/s.

VD: *G. elata* slices were evenly spread on a tray, and then placed in the vacuum drying oven (DZF-5050S, Kangheng Instruments, Guangzhou, China). The drying temperature was set to 60 °C and the vacuum degree was 0.095 MPa.

MD: *G. elata* slices were evenly spread in the center of the oven chamber and the drying power was set to 340 Watts (W) (NE-186AC, Panasonic, Osaka, Japan).

MVD: *G. elata* slices were evenly spread on a tray and the drying power was set to 340W (ORW1.OS-5Z, Aorun Instruments, Nanjing, China). The vacuum degree was 0.085 MPa.

FD: *G. elata* slices were pre-frozen at −80 °C for 24 h and then placed in a freeze dryer for drying (FD-1A-50, Yuming Instruments, Shanghai, China). The freezing trap temperature was set to −50 °C. 

Control: Unsteamed *G. elata* slices were evenly spread on a tray and then placed in the hot-air-drying oven at 60 °C. This group of samples was used as a control for the other steamed samples.

### 2.3. Drying Characteristics

#### 2.3.1. Drying Rate

HAD and VD samples were weighed every 1 h, while MD and MVD samples were measured every 2 min. The FD samples were weighed every 4 h. The change in weight was recorded until the difference in weight between the two measurements was less than 0.01 g, which was regarded as the termination of drying. The dry basis moisture content (Mt) and drying rate (DR) were calculated using Formulas (1) and (2) [17].

(1) $$M_{t}=\frac{m_{t}-m_{d}}{m_{d}}$$

where m_t_ is the real-time mass (g) and m_d_ is the absolute dry mass (g).

(2) $$\mathrm{DR}=\frac{M_{t+\mathrm{dt}}-M_{t}}{d_{t}}$$

where M_t+dt_ and M_t_ are the moisture content at time t + dt and t (g/g, dry basis), d_t_ is the time difference between the two measurements.

#### 2.3.2. Low-Field Nuclear Magnetic Resonance Measurement

The dried *G. elata* slices were placed in the center of magnetic coil. The free induction decay (FID) method was used for calibrating and adjusting the center frequency, and then the transverse relaxation time (T2) was determined by Carr–Purcell–Meiboom–Gill (CPMG) pulse sequence. The T2 inversion spectrum was then obtained using inversion software V4.0. The corresponding parameters for the CPMG were set as follows [18]: offset frequency (O1) = 383,203.9 Hz sampling frequency (SW) = 100 kHz, 90° pulse time (P1) = 12.52 μs, 180◦ pulse time (P2) = 25.52 μs, time echo (TE) = 0.3 ms, time waiting (TW) = 2500 ms, number of scan (NS) = 32, and number of echoes (NECH) = 20,000.

### 2.4. Structural Characteristics

#### 2.4.1. Scanning Electron Microscopy (SEM)

The surface tissues from the dried *G. elata* slices were gilded and observed by SEM-450 (FEI, Hillsboro, OR, USA) with 200× magnification. 

#### 2.4.2. Fourier Transform Infrared Spectroscopy (FTIR)

An amount of 1 mg of *G. elata* powder was mixed with 200 mg potassium bromide (KBr). The samples were scanned 32 times with a spectral resolution of 8 cm^−1^ and a scanning range set to 4000–400 cm^−1^

### 2.5. Physical Characteristics

#### 2.5.1. Color Analysis

A colorimeter (Ci7600, X-Rite company, Grand Rapids, MI, USA) was used to measure the color of *G. elata* powder. L* represents brightness, a* represents red and green, and b* represents yellow and green. Taking the group of unsteamed samples as the control, the overall changes in color ΔE were obtained from the Formula (3).

(3) $$\Delta E=\sqrt{{\left({\Delta L}^{*}\right)}^{2}-{\left({\Delta a}^{*}\right)}^{2}-{\left({\Delta b}^{*}\right)}^{2}}$$

#### 2.5.2. Particle-Size Analysis 

A particle-size analyzer (LS13320, Beckman Coulter, Brea, CA, USA) was used to determine the particle size and specific surface area of the *G. elata* powder samples. Each group of samples was measured three times and then the average value was recorded.

#### 2.5.3. Water-Holding Capacity and Oil-Holding Capacity

A 0.5 g of sample was placed into a 100 mL centrifuge tube with 20 mL of distilled water. The sample was then stirred well with a glass rod and centrifuged at 5000 r/min for 20 min. The supernatant was then discarded and the total mass of the centrifuge tube and precipitate was recorded. The determination of each group of samples was repeated three times to take the average value. The method of oil-holding capacity is the same as water-holding capacity, and the distilled water can be replaced by oil. 

### 2.6. Determination of Active Ingredients

The high-performance liquid chromatographic method for the determination of *G. elata* in the Pharmacopoeia of 2020, with slight adjustments, was used. A total of 2 g of *G. elata* powder was mixed with 50 mL of diluted ethanol and ultrasonicated for 30 min. The sample was then filtered and placed in an evaporating dish to dry. The residue was then dissolved with a mixture of acetonitrile and water, and then filtered through a 0.45 μm filtration membrane. The chromatographic (LC-20A, Shimadzu, Kyoto, Japan) conditions were as follows: C18 column (250 mm × 4.6 mm, 5 μm); mobile phase: acetonitrile-0.05% phosphoric acid solution (3:97); detection wavelength: 270 nm; detection temperature: 30 °C; injection volume: 10 μL; flow rate: 0.8 mL/min.

### 2.7. Flavor Characteristics

#### 2.7.1. Electronic Noise (E-nose) Analysis

Preheating and calibration were performed according to the E-nose (PEN3, Airsense Analytics GmbH, Schwerin, Germany) operating standards. A 5.00 g sample was massed into a 20 mL headspace vial and enriched for 30 min at room temperature before detection. 10 measurements were performed for each group of samples.

#### 2.7.2. GC-MS Analysis

For GC-MS (QP2010, Shimadzu, Japan) analysis, a 5 g sample was massed into a 20 mL headspace vial and 10 μL of octanol was added as an internal standard (10 mg/L). The chromatographic conditions were as follows: DB-17MS capillary column (60 m × 0.25 mm × 0.25 µm). The column temperature was set to 40 °C as the beginning column temperature and maintained for 3 min. It was then increased to 240 °C at a speed of 4 °C/min and maintained for 10 min. The inlet temperature was 250 °C; helium was used as the carrier gas at a flow rate of 1 mL/min. 

For the mass spectrometry conditions, the ionization temperature was set to 250 °C; electron energy 70 eV; scanning range 40~400 *m*/*z*. The results were matched with those from the NIST spectral libraries, and the names of the volatiles and their related information were determined with this library. The samples were then quantitatively measured by using the internal standards.

### 2.8. Statistics Analysis

Experimental data were analyzed using SPSS Statistics 26 and plotted using Origin 2024. E-nose data were analyzed by PCA with LDA using Winmuster 1.2.

## 3. Results

### 3.1. Drying Characteristics

**Figure 1:**
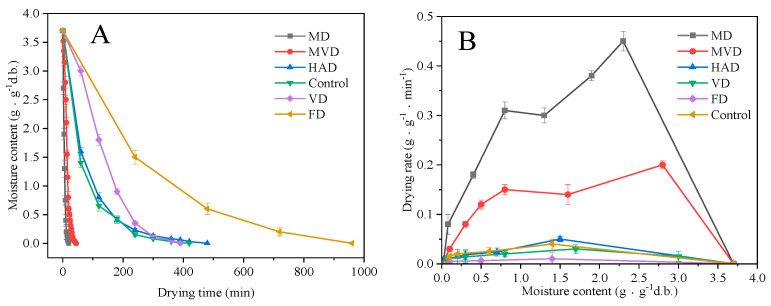
Drying−time curve (**A**) and drying−rate curve (**B**).

The initial dry-basis moisture content of the samples was 3.7 ± 0.08 g/g. The drying curves and drying rate curves under different drying methods are shown in Figure 1. The time to reach the drying endpoint for FD, HAD, VD, MD, MVD and control samples were 16 h, 8 h, 6.5 h, 20 min, 44 min, and 7 h, respectively. Among them, the drying time for the group of unsteamed samples that acted as our control was 1 h shorter than other steamed samples. The MD sample had the shortest drying time, followed by MVD, HAD, and VD samples, which were similar in drying time. Meanwhile, the FD sample had the longest drying time. Previously, similar results were found with dried Cistanche deserticola [19] and Bletilla striata flowers [20]. The drying curves of the different drying methods showed similar trends, where the moisture content in the early drying stage rapidly declined, then the rate of decline significantly reduced in the later stage until the completion of drying. With the increase in temperature and power, we found that the water migration accelerated, which shortened the drying time to increase the drying rate. This is consistent with the findings of Kutlu [21] and Horuz [22] in regard to dried bee bread and sour cherries.

Altogether, the drying rates of 6 drying methods showed a decreasing trend, and there was no obvious constant-rate drying stage. Consequently, this indicated that the diffusion and migration of water in the sample determined the drying rate. The drying rates for MD and MVD methods were significantly higher than they were for the other samples, since the heat source was microwaves that directly penetrate the sample. The microwave energy can be rapidly absorbed by water molecules to achieve the effect of balanced internal and external heating, thus having the same heat and mass-transfer direction, which is more conducive to the evaporation of water [19]. Compared with MD, the vacuum condition in the MVD method did not expose the samples to high temperature, better retaining the original shape and color of the sample and acting as an efficient and high-quality drying technique.

### 3.2. Effect of Drying on Water Status

The relaxation time (T2) and the signal amplitude (A2) were determined by LF-NMR, which reflects the chemical environment of hydrogen protons and the degree of water freedom in drying *G. elata* samples. Typically, the smaller the T2, the closer the combination of water and non-aqueous components, which produces less water freedom and causes the peak position to shift more to the left. The water in the dried *G. elata* samples can be divided into bound water T21 (1~10 ms), immobile water T22 (10~100 ms), and free water T23 (100~1000 ms). Among them, bound water can be further divided into strongly bound water T20 (0.01~1 ms) and weakly bound water T21 (1~10 ms). Here, we found that A21, A22, and A23 were the relative peak areas at the corresponding times, which represented the relative content of water in their corresponding state [23].

**Figure 2:**
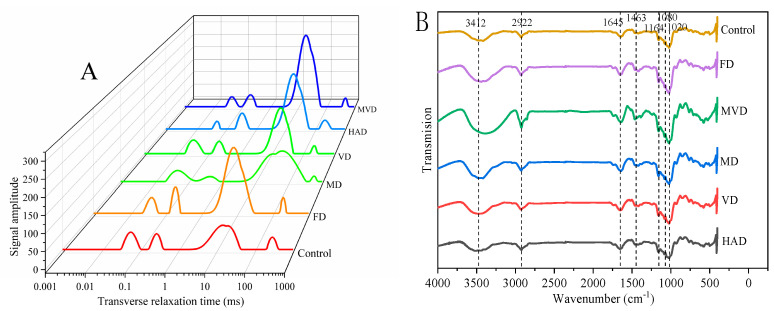
T2 inversion spectra (**A**) and FT−IR spectra (**B**).

Figure 2A shows the T2 inversion spectra of *G. elata* with different drying methods. The dried *G. elata* slices contained more immobile water and less bound water, while the free water was mostly evaporated and had diffused. Together, this indicates that free water was the most mobile, and had the lowest binding ability that was easiest to remove with drying. With the MD sample, some peaks disappeared, while some were newly formed. This may be because the microwave destroyed the internal tissue of the sample and there was no clear boundary between bound water, immobile water, and free water. In comparison, this same result was found in dried carrot [24]. With the different drying methods, the small range of variation in T21 and T23 indicates that the bound water is tightly bound to macromolecules and is not easily removed during the drying process. Additionally, the amplitude of T22 varies to different degrees with different drying methods, which may be due to the disruption of cell membranes during the drying process and the influx of macromolecules. The macromolecules include compounds, such as polysaccharides, that move into the cellular interstitial space, which increases the concentration of poor mobile water and macromolecule binding opportunities [25]. Ultimately, the less mobile water is the main water in the *G. elata* sample left at the end of drying.

### 3.3. Apparent Morphology and Microstructure

**Figure 3:**
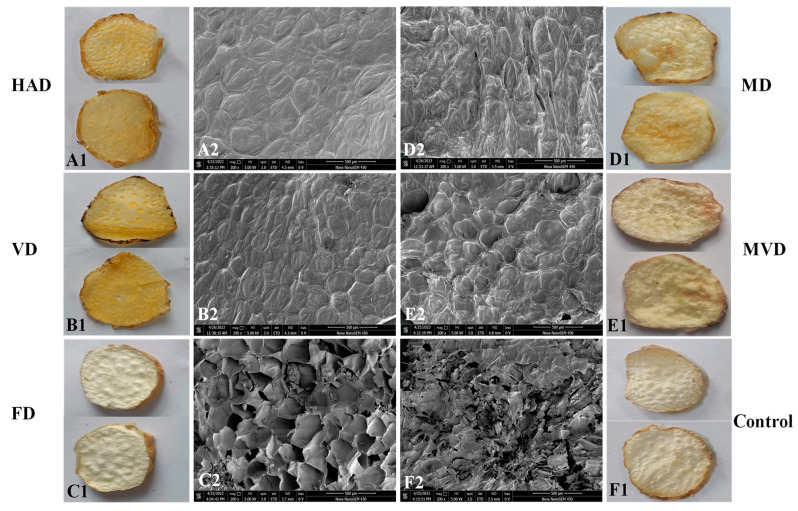
Appearances (**A1**–**F1**) and SEM images (**A2**–**F2**).

The apparent morphologies of the *G. elata* samples exposed to different drying methods are shown in Figure 3. The MD and MVD samples possessed pores on their surface, which produced internal hollowing and blistering, and localized scorching from the inhomogeneity of the microwave. Additionally, the MD samples rapidly lost moisture during drying, and the quick contraction of the surface caused uneven distribution of moisture, so that incomplete local drying occurred. Except for the FD method, all the drying methods caused curling and shrinkage, these effects being most serious in the group of unsteamed samples. Consequently, it can be seen that FD maintains the original morphology and color of the samples better, followed by MVD.

SEM images also reflect the effects of different drying methods on the microstructure of *G. elata*. The images of the unsteamed samples and other steamed samples were obviously different. The cell structure of the unsteamed samples was clear, and the cell wall ruptured by water loss after drying. Meanwhile, the other samples went through enzyme destruction through steaming, so that the cell tissues swelled after adsorbing water. Previously, Xie et al. [9] found that when the central temperature of a *G. elata* sample exceeded 55 °C during steaming, sticky fragments were formed in the cells due to the hydrolysis of polysaccharides. Further, it was difficult for the moisture loss to be migrated. Therefore, the drying time of the samples was prolonged by steaming.

Ultimately, this view has also been confirmed in the context of the other drying properties. For example, the HAD method resulted in samples with a compact and intact cellular structure. Likewise, the VD samples exhibited a similar appearance with more tightly arranged cells under the dual effects of temperature and pressure. Meanwhile, the MD and MVD samples showed obvious fractures and holes on the surface, with severely damaged and disordered cellular structures, probably due to microwave disruption of cellular tissues. The FD samples showed uniform honeycomb pores due to the sublimation of ice crystals into water vapor, which left voids with a sponge-like porous structure in the cells [26]. In general, other reports have also suggested that these microscopic cavities could enhance the content of active ingredients extracted from the samples, which led us to analyze differences in active compounds [27].

### 3.4. FT-IR Spectra

The effects of the different drying methods on the main functional groups within the molecules of *G. elata* powder were analyzed by Fourier infrared spectroscopy (FT-IR). Our results are shown in Figure 2B. It can be seen that the infrared spectra of the different dried samples mainly consisted of single peaks with similar position and shape, but the peak intensity is different. The chromatographic peaks of *G. elata* samples under different drying methods were more strongly absorbed at 3412 cm^−1^, 2922 cm^−1^, 1645 cm^−1^, 1463 cm^−1^, and 1020 cm^−1^. The peak intensity of the MVD samples was higher than that of the other samples, with the unsteamed samples having the lowest peak intensity. The remaining samples had little difference in the intensity of the characteristic peaks at each location. In addition, the broader and stronger absorption peak at 3412 cm^−1^ was caused by the stretching vibration of O-H, which is formed by hydrogen bonding due to the formation of hydrogen bonding by the hydroxyl groups in the molecules of *G. elata* glycosides. Likewise, symmetric and asymmetric stretching vibrations for C-H bonds by methyl and methylene groups form an absorption peak at 2922 cm^−1^. Classically, the presence of an absorption peak near 1645 cm^−1^ is caused by the stretching vibration of C=O. The absorption peak at 1418 cm^−1^ consists of a stretching vibration of C-N probably caused by the protein. Further, the stretching vibration of the C-O bond within the polysaccharide produces three moderately strong absorption peaks near 1200–1000 cm^−1^ that are sequentially enhanced. It can be seen that the positions of the spectral peaks of the different samples are similar, as well as the peak shapes, and that there are only differences in absorption strength. Together, this data indicates that the structure of *G. elata* from the different drying methods is similar, which did not cause unique changes in the internal structure, and its main components did not undergo fundamental changes with different drying. Previously, the FTIR spectra of daylily powder [28] and red sea bream surimi powder [29] were not changed after different drying methods, which is similar to our data.

### 3.5. Color Analysis

The color changes of the *G. elata* powder samples exposed to different drying methods are shown in Table 1. Taking the group of unsteamed samples as the control, the color variation among the samples was significant. The FD samples had the largest L* value, followed by the MVD samples. Most likely, this was because they were both in a vacuum environment, and the drying temperature for the FD treatment was low while the drying time for the MVD treatment was short, which prevented enzymatic and non-enzymatic browning from occurring.

The FD samples also had the highest brightness and the lowest amount of red and yellow, which indicates that this drying method better retains the original color of the samples. The VD samples had the smallest L*, and the largest a* value and b*, followed by MD. Together, this might be due to the fact that when the temperature and power are too high, it is easy to cause a local hot spot, which leads to local scorching or browning of the sample, thus making the powder yellowish in color with lower brightness and greater color variation [30].

In addition to these parameters, the ΔE value indicates the chromaticity difference between the samples. Here, we found that the ΔE value for the MVD samples was significantly lower than that of other samples, which indicates that the samples treated by microwave vacuum have the least change in chromaticity. Together, this implies that the MVD method can better reduce the influence of temperature with chromaticity. Xu et al. [31] also found a similar result using MVD, which was carried out in with light avoidance and vacuum conditions. They also found that, to a certain extent, MVD can reduce the color loss of lemon slices with minimum color-difference values.

### 3.6. Particle-Size Analysis

The particle-size distribution of *G. elata* powder is shown in Table 2. Typically, D50 is the median particle size, and indicates the average particle size of the powder. The span indicates the uniformity of particle-size distribution, and a larger the span usually signifies more dispersion with larger particle size [32]. In addition, specific surface area indicates the particle size of the powder, and a larger specific surface area usually corresponds to smaller powder particles.

The dried samples were subjected to the same pulverization process but showed significant differences in particle size. The FD samples had the smallest average particle size, followed by the MD samples. This may be due to the fact that the moisture in the samples during the FD process directly sublimates from the ice-crystal state to water vapor, which makes the internal structure loose and porous and easy to pulverize. In the MD process, however, the water evaporates quickly to form more pores and the drying time is short, which is favorable for pulverization. In addition, we found that the dispersion of the HAD samples was the largest, followed by the VD samples. This may be due to the longer drying time and higher temperature associated with the HAD and VD methods, which make the surface of the sample harden and shrink. After drying, the sample organization is dense, which is not conducive to uniform pulverization.

In addition to these parameters, the specific surface areas of the FD samples and the unsteamed samples were significantly larger than that of the other samples, with the MD samples having the second-largest surface areas. Together, this implies that under the same drying conditions, the specific surface area of an unsteamed sample is much larger than that of a steamed sample. However, the two sample groups have a similar span, which signifies that steaming will make the powder particle size larger without significantly changing the degree of dispersion. Ultimately, this may be due to the formation of viscous segments in the cells of the steamed samples, which makes it difficult for water to migrate and causes the drying time to be longer than that of the unsteamed samples. This idea has also been confirmed with microstructure data.

### 3.7. Water-Holding Capacity (WHC) and Oil-Holding Capacity (OHC)

The effect of different drying methods on water-holding capacity (WHC) and oil-holding capacity (OHC) on *G. elata* powder is shown in Table 2. Higher WHC helps to reduce water loss from the food. Higher OHC stabilizes the food form, reduces the loss of fats and oils, and slows down the rate of oxidation and spoilage. Here, we found the different drying methods significantly affected WHC and OHC to different degrees. The WHC of the FD samples and the unsteamed samples were much lower than that of the others, which may be attributed to the fact that these two samples have small powder particle sizes and have a weaker ability to bind water. Meanwhile, the MVD samples had the highest WHC, and there was no significant difference in the WHC with the other samples.

With OHC, the FD samples had the highest capacity, followed by the unsteamed samples. There was no significant difference in the OHC for the other samples. This may be due to the large specific surface area of these two samples, which can adsorb more oil in the same volume. In addition, the other drying methods could destroy the protein structure of the samples, to some extent, with the higher temperatures not being favorable for the adsorption of oil and grease by the powder. Likewise, under the same drying conditions, the WHC of the unsteamed samples is much smaller than that of the steamed samples, while the OHC is similar. Together, these data indicate that steaming favors a significant increase in the WHC of *G. elata* powder but has no significant effect on the OHC. This may be due to the fact that heat treatment leads to starch pasting and swelling for crude fibers, which results in an increase in water absorption [33]. Similar conclusions were also obtained by Sanchiz et al. [34], who found that the WHC and OHC of their pistachio and cashew samples increased after moist-heat treatment.

### 3.8. Active Ingredients

With active ingredients, several chemicals have been identified in *G. elata* that are known to cause physiological effects. For example, gastrodin (GA) is known to cause sedation, and acts as an anticonvulsant [35]. It has also been found to increase intelligence and brain health, as well as delay aging and enhance immunity. Likewise, p-hydroxybenzyl alcohol (HA) has a protective effect on the nervous system, while p-hydroxy benzaldehyde (HBA) is a precursor for gastrodin and has anti-platelet aggregation effects. Together, they act as the main active ingredients in *G. elata* that can be used as an important index to evaluate the quality standard for *G. elata*.

The effects of different drying methods on the active ingredients of the *G. elata* powdered sampled are shown in Table 3. Typically, the β-glucosidase in fresh *G. elata* will make gastrodin decompose, so it should be steamed before drying, and the high temperature inactivates the enzyme while promoting the conversion of some of the active ingredients in *G. elata*. Under the same drying conditions, the gastrodin content of the steamed samples was greater than that of the unsteamed samples. Meanwhile, the p-hydroxybenzyl alcohol and p-hydroxybenzaldehyde contents were lower, and the total content was 1.71 times higher.

All the different drying methods produced samples with a gastrodin content that was generally higher than the p-hydroxybenzyl alcohol and p-hydroxy benzaldehyde contents, which may be due to the fact that the interaction of p-hydroxybenzyl alcohol with sugar produces gastrodin [36]. Of the several drying methods, the FD samples had the highest gastrodin content, followed by the MVD samples. This may be due to the fact that the physiological and biochemical effects of the FD samples were terminated in the frozen state, which prevented the degradation of gastrodin and preserved the active ingredients. Additionally, the MVD method most likely reduced water in a short period of time from the samples’ heat-sensitive components. At the same time, the microwave penetration was strong, which is conducive for the rapid dissolution of the active ingredients. Our results are also similar to those from a previous study by Fan et al. [37], which also confirmed that the microwave treatment could increase the content of gastrodin, p-hydroxybenzyl alcohol, and other active components in *G. elata.*

### 3.9. E-Nose Analysis

**Figure 4:**
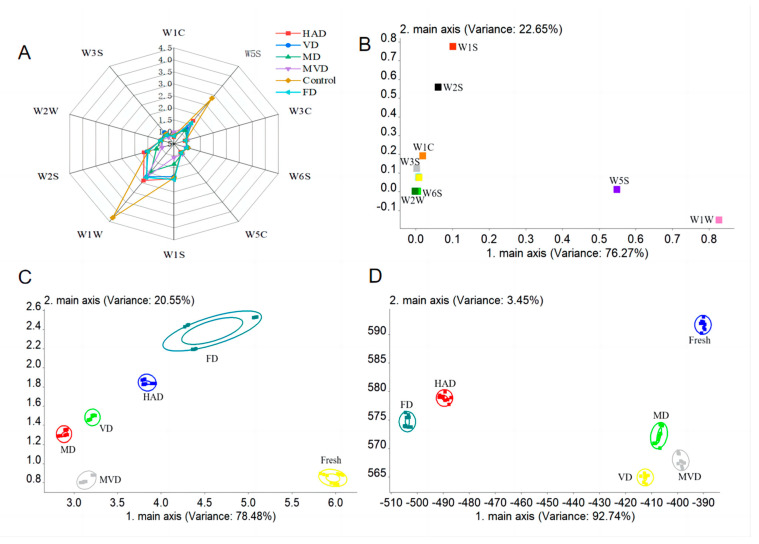
Effects of different drying methods on E-nose responses of *G. elata* powder. Sensor response radar diagram (**A**); component distribution plot (**B**); PCA sample distribution diagram (**C**); LDA sample distribution diagram (**D**).

In our analysis, electronic nose (E-nose) consists of 10 sensors through which volatile compounds are recognized for further classification within a sample. Figure 4A shows E-nose radargram, and that the response values for the W5S (nitrogen oxide sensitive), W1W (sulfide sensitive), W2S (alcohol sensitive), and W1S (methyl sensitive) sensors were significantly higher than those for the other sensors. In particular, the response values for two sensors, W1W and W5S, were significantly higher for the unsteamed samples compared to the other samples. This suggests that the unsteamed samples are the richest in odor content, and that steaming causes the loss of volatile compounds. The sensor distribution plot in Figure 4B reconfirms the difference in response among the different sample sensors, and shows that W1S and W1W have the highest response followed by W5S and W2S. Together, this suggests that they are the main sensors that distinguish the *G. elata* samples according to their different drying methods.

In order to further assess the differences in volatile compounds among the samples, we performed PCA and LDA analysis of the E-nose data. PCA performs data downscaling on the response signals, whereas LDA accounts for the class of the sample based on all the response signals [38]. In Figure 4C, the contribution of PC1 and PC2 were 78.48% and 20.55%, respectively, with a total contribution of 99.03%, which was able to express the different *G. elata* samples well. There was no overlap between the samples, which suggests that the *G. elata* samples exposed to different drying methods all had unique flavor components. The unsteamed samples and FD samples were farther away from the other samples, which implies that steaming and low temperatures change the content and type of volatile compounds in *G. elata*. Meanwhile, the VD and MD samples were very close to each other, which suggests that the overall difference in volatile composition of the samples was relatively small and share commonalities. In Figure 4D, the contributions of the linear discriminant functions, LD1 and LD2, were 92.74% and 3.45%, respectively, with a total contribution of 96.19%. The samples can all be distinguished, which was consistent with the results from our PCA analysis. Together, these data imply that there are some differences in volatile components between the PCA and LDA analysis. Together, these results are similar to those of different drying methods that were investigated for ginger [39]. However, the reason for the differences may be the formation of new volatile compounds with the different drying conditions [40].

### 3.10. GC-MS Analysis

HS-SPME-GC-MS was used to analyze the volatile components in *G. elata*. Initially, we selected compounds with similarities higher than 85% for comparison with the NIST library, and the results are shown in Table 4. A total of 36 volatile substances were detected, which were categorized into four types: aldehydes (10), alcohols (6), hydrocarbons (15), and other types of compounds (5). In the unsteamed samples, we identified 24 substances, including 6 aldehydes, 5 alcohols, 9 hydrocarbons, and 4 other types of compounds. Meanwhile, the different drying methods affected the types of volatile components. In the HAD, VD, MD, MVD, and FD samples, we identified 21 (7 aldehydes, 1 alcohol, 11 hydrocarbons, and 2 other types of compounds), 22 (7 aldehydes, 2 alcohols, 11 hydrocarbons, and 2 other types of compounds), 23 (7 aldehydes, 1 alcohol, 12 hydrocarbons, and 3 other types of compounds), 27 (7 aldehydes, 2 alcohols, 15 hydrocarbons, and 3 other types of compounds), and 19 (5 aldehydes, 2 alcohols, 11 hydrocarbons, and 1 other type of compounds).

With volatiles, the unsteamed sample had the highest total content of 227.22 mg/mL, followed by the MVD samples (137.26 mg/mL). In comparison, the HAD samples (104.33 mg/mL) and VD samples (111.65 mg/mL) had the lowest total volatiles content.

In addition, aldehydes and alcohol compounds contributed more to the volatiles in *G. elata*. Compared with other samples, the highest aldehyde contents were found in the unsteamed sample (77.46 mg/mL), whereas the FD sample (50.70 mg/mL) had the highest retention, probably due to the high temperature that inactivated lipoxygenase, which oxidizes unsaturated fatty acids as the main pathway for aldehyde formation and inhibits the production of aldehydes [41]. Among them, n-hexenal was the most abundant aldehyde, producing a fruity and grassy aroma in both the unsteamed (45.64 mg/mL) and FD (25.02 mg/mL) samples. Likewise, trans-2-octenal was another volatile substance unique to the FD samples, bringing a characteristic fatty aroma to the samples. However, 2-methylbutyraldehyde, benzaldehyde, and glacial acetic acid did not appear in the FD samples, which indicates that freeze-drying is effective in eliminating irritating odors in *G. elata*. Interestingly, the unique 1-octen-3-ol, 3,5-octadien-2-ol, and phytol were only detected in the unsteamed samples, creating the mushroom aroma.

In regard to hydrocarbons in these samples, they mainly included alkanes, olefins, alkynes, and naphthalenes. The hydrocarbons mainly originated from the breakage of fatty-acid alkoxy radicals, which had a high flavor threshold and contributed less to the overall flavor of *G. elata*. The main hydrocarbons in the samples were n-dodecane and decahydro-2,6-dimethylnaphthalene, which have waxy and tarry flavors. In addition, limonene was present in all samples at low levels and created a distinctive orange flavor that contributed to the overall flavor.

Among the other classes of compounds, n-hexanoic acid (51.95 mg/mL) and p-cresol (25.52 mg/mL) were the unique volatile components in the unsteamed samples and the main source of this group’s irritating odor. Localized hot spots occurred during MD and MVD due to the uneven microwave, which led to the scorching phenomenon in the *G. elata* samples and the generation of volatile flavor compounds, like 2-ethylfuran, that cause a burnt odor. In addition, 2, 3, 5, 6-tetramethylpyrazine also has a musty odor, which further confirmed that this substance might be the cause of the special odor (horse urine odor) in *G. elata*.

**Figure 5:**
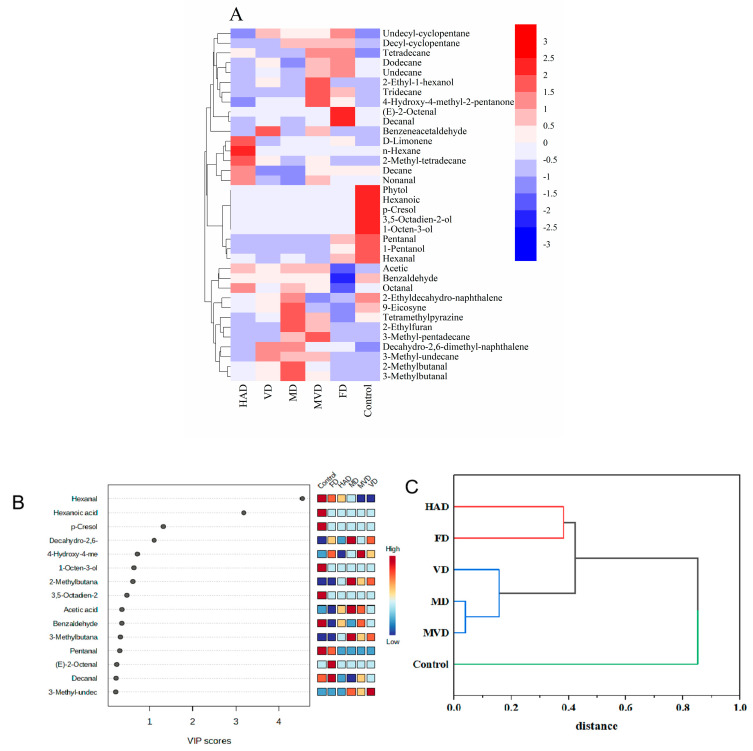
Multivariate statistical analysis of different drying method by HS-SPME-GC-MS identified volatiles on *G. elata* powder. Heatmap cluster based on the normalized quantities of the identified volatiles (**A**); VIP scores in PLS−DA (**B**); Cluster analysis diagram (**C**).

A total of four differential signature flavor substances were screened out from these volatiles using the magnitude of the VIP value from our partial least squares (PLS-DA) model. Here, substances with VIP >1 are usually considered as important and potential chemical markers for the study of the model. The VIP scores for the volatile compounds in asparagus are shown in Figure 5B. These substances may be the main volatile flavor substances responsible for the flavor differences resulting from the different drying methods for *G. elata*. Among these four volatile substances, there was one aldehyde (hexanal), one hydrocarbon (decahydro-2,6-dimethylnaphthalene), and two acids (hexanoic acid, p-cresol). Between them, hexanal showed the highest VIP value among all the samples. Together, these data indicate that aldehydes are important components that affect the differences between different dried samples. At the same time, they also imply that the diversity of volatile compositions among different dried samples is mainly attributed to fatty acid changes caused by lipid degradation reactions.

To further confirm the differences between the samples, all volatile components were analyzed with cluster analysis, and the results are shown in Figure 5C. The clustered class I includes HAD and FD samples, while class II includes VD, MD, and MVD samples, and class III included the unsteamed samples. Ultimately, these data suggest that the volatile components of the unsteamed samples were significantly different to those of the other samples, and that the HAD samples had similar flavors to the FD samples, while the VD, MD, and MVD samples had similar flavors. This may be due to the fact that the VD, MD, and MVD samples all underwent a Maillard reaction at high temperatures that produce similar volatile products. There were also partial similarities in the volatiles in HAD and FD samples, such as n-hexanal and n-decanal, which suggests that the odors produced from these two drying methods would be partially similar. Altogether, these data are also consistent with the E-nose PCA and LDA analysis results.

Ultimately, our data show that the changes in flavor substances are mainly caused by the degradation and oxidation of lipids, Maillard reactions, and enzymatic reactions [42]. Aldehydes, alcohols, and hydrocarbons were the main substances responsible for the flavor variation among *G. elata* samples. In addition, we found that the time and temperature of the drying process affects flavor changes. By comparing the flavor substances of the different drying methods, we found that the FD method effectively eliminated irritating odors in *G. elata*, while the MVD method had the most volatile substances and the strongest aroma. Different drying methods have different rates of water loss, and both the degree and the rate of the reduction in moisture content are factors that affect the rate of these reactions. Still, our work shows that drying methods such as FD or MVD would be beneficial to use for the industrial growth of *G. elata* because of their specific effects on these flavor compounds.

## 4. Conclusions

In summary, the type of drying method used produces significantly different effects on the quality and flavor characteristics of *G. elata*. Our results show that freeze drying (FD) had the longest drying time, while microwave drying (MD) and microwave vacuum drying (MVD) had the shortest drying times. FD also best maintained the appearance and color of *G. elata*, and maintained the highest concentration of active ingredients, followed by MVD. In addition, we also found that under the same drying conditions, steaming significantly increased the water-holding capacity, oil-holding capacity, and active ingredients. Our electronic nose data also showed that W1W and W1S were the main sensors that distinguish the flavor profiles of *G. elata*. Further, our HS-SPME-GC-MS analysis detected 36 volatile compounds, with the most species identified in the MVD samples. Likewise, the unsteamed samples had the highest water content, whereas FD was effective at eliminating any pungent odors from *G. elata*. Therefore, these findings suggest that the FD method can achieve the best appearance and color of *G. elata*, while MVD greatly improves its drying efficiency. Considering the inherent quality and actual cost factors associated with *G. elata*‘s industrial preparation, the MVD method is recommended because it is the most cost effective. Ultimately, this study provides a theoretical basis for the drying and processing of *G. elata*, while promoting the further development and utility of *G. elata*.

## Figures and Tables

**Table 1:** Effects of different drying methods on color of *G. elata* powder ^1^.

Samples	L*	a*	b*	ΔE
Control	88.25 ± 0.21 ^c^	1.57 ± 0.01 ^c^	9.58 ± 0.04 ^e^.	-
HAD	86.74 ± 0.01 ^d^	1.22 ± 0.02 ^e^	11.63 ± 0.03 ^c^	2.55 ± 0.03 ^d^
VD	84.45 ± 0.01 ^e^	2.34 ± 0.02 ^a^	12.75 ± 0.01 ^a^	5.05 ± 0.01 ^a^
MD	86.80 ± 0.01 ^d^	1.82 ± 0.01 ^b^	12.66 ± 0.05 ^b^	3.41 ± 0.04 ^b^
MVD	88.61 ± 0.02 ^b^	1.38 ± 0.02 ^d^	11.26 ± 0.01 ^d^	1.79 ± 0.05 ^e^
FD	91.03 ± 0.02 ^a^	0.35 ± 0.00 ^f^	8.59 ± 0.04 ^f^	3.22 ± 0.02 ^c^

^1^ Data are expressed as mean ± standard deviation. Means with different letters within the same column are significantly different (*p* < 0.05).

**Table 2:** Effects of different drying methods on particle-size distribution, water-holding capacity (WHC), and oil-holding capacity (OHC) of *G. elata* powder ^1^.

Samples	D10	D50	D90	Specific Surface Area (cm^2^/g)	Span	WHC (g/g)	OHC (g/g)
HAD	18.65 ± 0.35 ^a^	98.00 ± 8.49 ^c^	289.50 ± 3.54 ^a^	2432.5 ± 34.7 ^e^	2.78 ± 0.21 ^a^	9.42 ± 0.30 ^b^	2.07 ± 0.06 ^c^
VD	15.05 ± 0.49 ^c^	98.80 ± 2.12 ^bc^	289.00 ± 5.66 ^a^	2947.0 ± 73.6 ^d^	2.77 ± 0.01 ^a^	9.37 ± 0.36 ^b^	1.99 ± 0.11 ^c^
MD	13.75 ± 0.64 ^d^	93.70 ± 2.69 ^c^	267.00 ± 5.66 ^b^	3226.0 ± 46.7 ^c^	2.71 ± 0.15 ^ab^	9.80 ± 0.23 ^b^	2.11 ± 0.06 ^c^
MVD	17.10 ± 0.71 ^b^	115.50 ± 3.53 ^a^	300.50 ± 13.44 ^a^	2912.0 ± 76.4 ^d^	2.46 ± 0.04 ^bc^	11.38 ± 0.38 ^a^	2.21 ± 0.08 ^bc^
FD	7.15 ± 0.11 ^f^	71.20 ± 0.56 ^d^	175.50 ± 3.54 ^c^	4863.5 ± 65.8 ^a^	2.37 ± 0.04 ^c^	3.66 ± 0.18 ^c^	2.55 ± 0.18 ^a^
Control	10.70 ± 0.28 ^e^	108.50 ± 2.12 ^ab^	292.50 ± 6.36 ^a^	4297.5 ± 47.4 ^b^	2.60 ± 0.01 ^abc^	2.22 ± 0.08 ^d^	2.37 ± 0.04 ^ab^

^1^ Data are expressed as mean ± standard deviation. Means with different letters within the same column are significantly different (*p* < 0.05).

**Table 3:** Effects of different drying methods on active ingredients of *G. elata* powder ^1,2^.

Samples	GA(mg/g)	HA(mg/g)	HBA(mg/g)
HAD	2.21 ± 0.26 ^a^	0.67 ± 0.41 ^a^	0.177 ± 0.028 ^a^
VD	1.53 ± 0.16 ^b^	0.47 ± 0.23 ^a^	0.089 ± 0.025 ^b^
MD	1.53 ± 0.25 ^b^	0.15 ± 0.10 ^a^	0.024 ± 0.010 ^c^
MVD	2.32 ± 0.30 ^a^	0.35 ± 0.16 ^a^	0.029 ± 0.009 ^c^
FD	2.71 ± 0.37 ^a^	0.47 ± 0.20 ^a^	0.019 ± 0.009 ^c^
Control	1.34 ± 0.06 ^b^	0.37 ± 0.08 ^a^	0.076 ± 0.016 ^b^

^1,2^ GA, gastrodin; HA, p-hydroxybenzyl alcohol; HBA, p-hydroxy benzaldehyde. ^b^ Data are expressed as mean ± standard deviation. Means with different letters within the same column are significantly different (*p* < 0.05).

**Table 4:** HS-SPME-GC–MS identified volatile components’ contents in *G. elata* powder at different drying methods.

Compounds	CAS	Contents (mg/mL)					
		HAD	VD	MD	MVD	FD	Fresh
aldehydes							
3-Methylbutanal	590-86-3	1.06	2.33	6.03	2.02	-	-
2-Methylbutanal	96-17-3	2.16	5.55	11.19	2.21	-	-
Hexanal	66-25-1	4.63	-	4.39		25.02	45.64
Octanal	124-13-0	3.26	1.39	2.59	2.19	-	1.84
Benzeneacetaldehyde	122-78-1	-	2.29	-	1.45	-	-
Benzaldehyde	100-52-7	12.03	11.63	11.59	12.60	-	13.04
Nonanal	124-19-6	15.50	7.34	6.70	13.71	8.82	10.35
Decanal	112-31-2	2.89	3.17	2.85	3.22	7.87	3.30
Pentanal	110-62-3	-	-	-	-	2.45	3.30
(E)-2-Octenal	2548-87-0	-	-	-	-	6.54	-
Subtotal (number)		7	7	7	7	5	6.00
Subtotal (content)		41.52	33.69	45.34	37.40	50.70	77.46
Alcohols							
4-Hydroxy-4-methyl-2-pentanone	123-42-2	6.07	12.34	11.83	22.60	15.69	8.32
2-Ethyl-1-hexanol	104-76-7	-	0.73	-	3.18	-	-
1-Pentanol	71-41-0	-	-	-	-	0.32	0.90
1-Octen-3-ol	3391-86-4	-	-	-	-	-	10.42
3,5-Octadien-2-ol	69668-82-2	-	-	-	-	-	7.72
Phytol	150-86-7	-	-	-	-	-	1.39
Subtotal (number)		1	2	1	2	2	5
Subtotal (content)		6.07	13.08	11.83	25.78	16.01	28.74
Hydrocarbons							
Undecane	1120-21-4	7.00	8.04	7.05	10.66	11.50	8.52
Dodecane	112-40-3	14.71	18.52	14.25	20.95	22.03	16.69
Tridecane	629-50-5	7.81	8.14	7.44	15.88	13.59	7.94
Tetradecane	629-59-4	1.91	1.15	1.07	2.66	2.59	0.42
Decane	124-18-5	2.92	-	-	1.54	1.67	1.90
3-Methyl-undecane	1002-43-3	-	2.02	1.89	1.76	-	-
Decyl-cyclopentane	1795-21-7	-	-	0.61	0.56	0.61	-
3-Methyl-pentadecane	19780-34-8	-	-	0.31	0.57	-	-
Undecyl-cyclopentane	6785-23-5	-	0.66	0.54	0.57	0.82	-
2-Methyl-tetradecane	1560-95-8	2.42	0.63	-	0.71	-	-
n-Hexane	110-54-3	3.33	-	-	0.43	-	-
D-Limonene	5989-27-5	3.85	0.69	0.98	0.97	1.59	0.70
9-Eicosyne	71899-38-2	2.54	2.93	4.14	2.39	1.86	3.27
Decahydro-2,6-dimethyl-naphthalene	1618-22-0	2.99	14.30	16.78	5.71	6.81	1.96
2-Ethyldecahydro-naphthalene	1618-23-1	1.55	2.07	2.58	0.99	1.23	2.50
Subtotal (number)		11	11	12	15	11	9.00
Subtotal (content)		51.03	59.16	57.63	66.36	64.31	43.89
Others							
Acetic acid	64-19-7	3.53	3.36	4.11	3.88	-	0.99
Tetramethylpyrazine	1124-11-4	2.17	2.36	3.76	3.14	1.65	2.67
2-Ethylfuran	3208-16-0	-	-	1.05	0.71	-	-
p-Cresol	106-44-5	-	-	-	-	-	21.52
Hexanoic acid	142-62-1	-	-	-	-	-	51.95
Subtotal (number)		2	2	3	3	1	4.00
Subtotal (content)		5.70	5.72	8.92	7.73	1.65	77.13
Total (number)		21	22	23	27	19	24.00
Total (content)		104.33	111.65	123.72	137.26	132.67	227.22

## Data Availability

The original contributions presented in the study are included in the article, further inquiries can be directed to the corresponding author.
